# P-1159. Likelihood of carbapenem-resistant Enterobacterales (CRE) importations into the United States: Understanding global colonization pressure and travel to inform public health

**DOI:** 10.1093/ofid/ofaf695.1352

**Published:** 2026-01-11

**Authors:** Velma K Lopez, Ashley R Styczynski, Matthew Westercamp, Fernanda C Lessa, Rachel Slayton

**Affiliations:** Centers for Disease Control and Prevention, Atlanta, Georgia; Centers for Disease Control and Prevention, Atlanta, Georgia; Centers for Disease Control and Prevention, Atlanta, Georgia; CDC, Atlanta, Georgia; Centers for Disease Control and Prevention, Atlanta, Georgia

## Abstract

**Background:**

A key risk factor for carbapenem-resistant Enterobacterales (CRE) acquisition among those without healthcare exposures is international travel. Individuals who are colonized with CRE may contribute to further CRE transmission, especially if hospitalized. Without CRE surveillance data among international travelers or data on health facilities admission post-travel in the United States (US), guidance for patient management is limited. However, modeling can provide insight into the likelihood of CRE importation (i.e. the likelihood that a CRE colonized person enters the US from elsewhere), which can be important in shaping the public health response to a newly emergent resistance mechanism.

**Figure 1:**
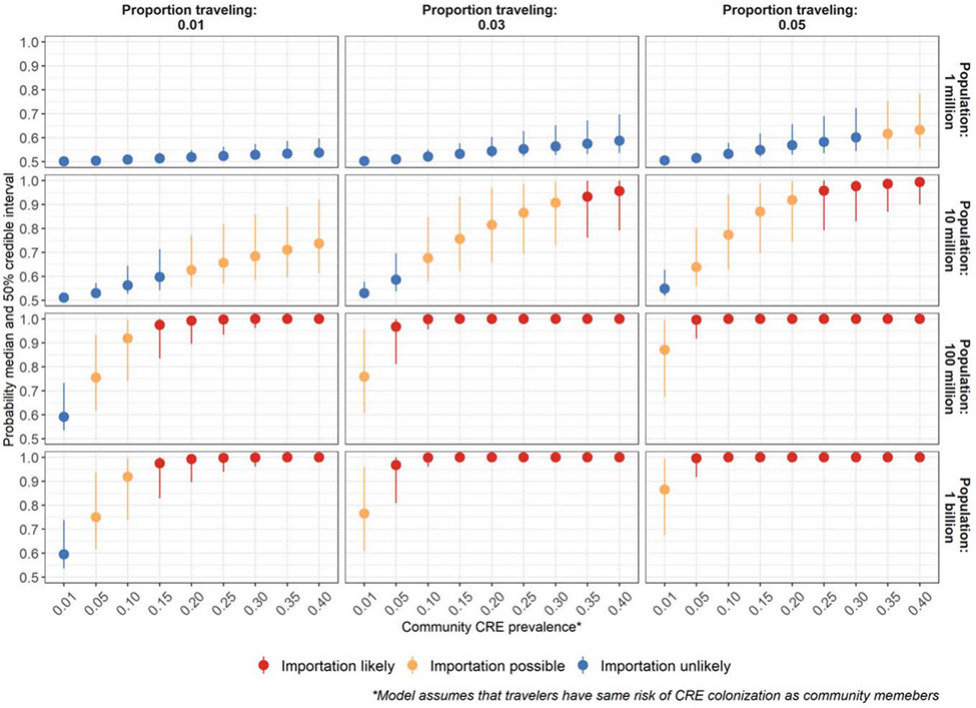
Probability of importation Median (as a point) and 50% credible interval (represented as a vertical bar) probability of CRE importation into the United States per simulated population. Each of the four simulations (presented as rows) uses a range of CRE community prevalence and travel volume parameters. Probabilities are color coded by the likelihood of an important event occurring.

**Methods:**

We simulated the annual, population-level probability of a CRE importation across four scenarios. Each scenario considered the source population (e.g. country) size, the travel volume from the source population, CRE community prevalence, and the duration of CRE colonization among travelers. We used a range of possible values for the source populations (1 million-1 billion people), CRE colonization prevalence (0.01-0.40, informed by the literature), and the proportion of the population traveling (0.01, 0.03, and 0.05, reflecting observed travel patterns); and sampled the duration of colonization (2 week minimum and median 3 months, informed by the literature).

We categorized the likelihood of an importation at the probability corresponding to quantile 0.25. We consider probabilities less than 0.50 as “unlikely”, between 0.50 and 0.75 as “possible”, and greater than 0.75 as “likely” to have occurred.

**Results:**

The probability of CRE importation increased as source population size, travel volume, and community CRE prevalence also increased (Figure 1). Regardless of travel volume, in large populations with CRE community prevalence > 15%, the probability of importation is nearly 1.0.

**Conclusion:**

The likelihood of CRE importation is high for those who traveled from countries with large populations and moderate CRE prevalence. While additional work is needed to estimate the impact of importations, these high probabilities suggest that global colonization pressure may play a role in the rise of US resistance mechanisms, which are often first recognized in healthcare settings.

**Disclosures:**

All Authors: No reported disclosures

